# Photoreactive Properties of Melanin Obtained from Human Induced Pluripotent Stem Cell-Derived Melanocytes

**DOI:** 10.3390/ijms26094119

**Published:** 2025-04-26

**Authors:** Krystian Mokrzynski, Mateusz Wojtala, Maciej Sulkowski, Shosuke Ito, Kazumasa Wakamatsu, Andrzej Zadlo, Marcin Majka, Tadeusz Sarna, Michal Sarna

**Affiliations:** 1 Department of Biophysics, Faculty of Biochemistry, Biophysics and Biotechnology, Jagiellonian University, Gronostajowa 7, 30-387 Krakow, Poland; krystian.mokrzynski@uj.edu.pl (K.M.); mateusz.wojtala@doctoral.uj.edu.pl (M.W.); andrzej.zadlo@uj.edu.pl (A.Z.); tadeusz.sarna@uj.edu.pl (T.S.); 2 Doctoral School of Exact and Natural Sciences, Jagiellonian University, Lojasiewicza 11, 30-348 Krakow, Poland; 3 Department of Transplantation, Faculty of Clinical Immunology and Transplantation, Institute of Pediatrics, Collegium Medicum Jagiellonian University, Wielicka 265, 30-663 Krakow, Poland; maciej.sulkowski@gmail.com (M.S.); mmajka@cm-uj.krakow.pl (M.M.); 4 Institute for Melanin Chemistry, Fujita Health University, Toyoake 470-1192, Aichi, Japan; sito@fujita-hu.ac.jp (S.I.); kwaka@fujita-hu.ac.jp (K.W.); 5 Department of Biophysics, Jagiellonian University Medical College, Sw. Lazarza 16, 31-530 Krakow, Poland

**Keywords:** melanin, eumelanin, melanocytes, hiPSC-Mel, physicochemical properties, photoreactivity, ROS, superoxide anion, singlet oxygen, EPR

## Abstract

Although melanin is viewed as a natural sunscreen that protects pigmented cells against the adverse effects of solar radiation, recent studies have demonstrated that, under certain conditions, the pigment can actually contribute to light-induced oxidative damage of the cells. However, the main issue with such studies is finding natural pigments without photooxidative modifications. Recently, melanin obtained from melanocytes, generated from human induced pluripotent stem cells (hiPSC-Mel), was suggested as a promising source of the pigment without significant photooxidation. Although different studies have demonstrated the feasibility of the above-mentioned technique to obtain melanin-producing cells, no thorough analysis of the physicochemical properties of the pigment has been performed. To address this issue, we examined the key physicochemical parameters, including the aerobic photoreactivity of melanin isolated from hiPSC-Mel and compared them with those of melanin from other known sources of the pigment, such as bovine retinal pigment epithelium (bRPE) and phototype V (PT-V) hair. Electron paramagnetic resonance (EPR) spectroscopy, dynamic light scattering, UV–Vis absorption and HPLC analysis of melanin degradation products were used. The ability of the examined melanins to photogenerate reactive oxygen species was determined by employing EPR oximetry, EPR spin-trapping and time-resolved singlet oxygen phosphorescence. Although the results of such measurements demonstrated that melanin obtained from hiPSC-Mel exhibited the physicochemical properties typical for eumelanin, a contribution from pheomelanin with a substantial presence of benzothiazine subunits, was also evident. Importantly, the hiPSC-Mel pigment had significantly lower photoreactivity compared to bRPE melanin and PT-V hair melanin. Our findings indicate that hiPSC-Mel could be an excellent source of high-quality pigment for photoprotection studies.

## 1. Introduction

Melanin is a natural pigment that in humans is mostly present in the skin, hair, retinal pigment epithelium, and dopamine-positive neurons [1,2,3,4]. Melanin in the skin is produced by specialized cells called melanocytes found in the basal layer of the epidermis [5,6,7]. The pigment is synthesized in the form of nanoaggregates that are stored in lysosome-related organelles called melanosomes [1,8,9]. Once melanin is synthesized, the organelles are transferred to neighboring keratinocytes where they form a supra-nuclear cap protecting the cells against solar radiation and other oxidative stress-inducing agents [10,11,12]. Even though melanin, especially the brown–black eumelanin, is generally viewed as a photoprotective pigment, it exhibits residual photochemical reactivity and can photogenerate reactive oxygen species, including superoxide anion and singlet oxygen [13,14,15,16,17]. Based on such observations, it was even postulated that melanin may contribute to the development of melanoma—one of the most aggressive types of cancer [18,19,20]. Indeed, some studies have indicated the role of melanin in the induction of melanoma by ultraviolet A (UVA) [21,22,23,24] that was associated with DNA damage in melanocytes, including the formation of cyclobutene pyrimidine dimers (CPDs), single strand breaks and 8-oxo-7,8-dihydroguanin (8-oxoGua) [25,26,27,28,29,30].

However, to address these issues experimentally, the selection of the appropriate melanotic systems is of importance. Thus, even though synthetic melanins are often used as convenient models of melanin pigments, their physical and photochemical properties might not accurately represent these properties in naturally occurring pigments. On the other hand, the availability of natural pigments in their native form, particularly of human origin, is a major problem, as the existing sources are either insufficient, i.e., skin biopsies that might provide only a limited amount of the pigment, or the isolated pigment might be substantially dehydrated (e.g., hair) or partially photodegraded (e.g., retinal pigment epithelium). The lack of an adequate supply of good quality natural melanin is a limiting factor impeding the research of photoreactive and photoprotective properties of skin melanin and its complete biological role. A possible solution is in vitro culture of pigmented cells derived from differentiated pluripotent cells that develop into functional melanocytes. It is expected that melanin obtained from such cells will be of superior quality and could be obtained in high quantity, compared to other sources of natural melanin, allowing thorough analysis of its physical properties and photochemical reactivity. It is known that the ability of melanins to generate reactive oxygen species (ROS) is based on the type of the pigment and the degree of photooxidative modifications that have accumulated in the pigment over its lifetime [14,31,32]. However, the fact that most natural melanins are subjected to differing exposures to sun light, which can induce oxidative modification of the melanin, may hinder the determination of the key physicochemical properties of natural melanins in their most native form [33,34]. Recently, melanocytes generated from human induced pluripotent stem cells (hiPSC) were suggested as a promising source of melanin with minimal effects from environmental factors [35,36,37,38,39]. Such cells can be obtained under fully controlled culturing conditions that minimize the exposure of the cells to UV and short-wavelength visible light. However, it remains unclear whether such melanin from human induced pluripotent stem cells (hiPSC-Mel) exhibits the physicochemical properties typical of other natural melanin pigments and, in particular, what is the aerobic photoreactivity of the melanin.

In this study, we examined the photoreactive properties hiPSC-Mel and compared them with melanins obtained from bovine retinal pigment epithelium (bRPE) and phototype V (PT-V) hair. As a control, we used synthetic DOPA-melanin. The obtained results demonstrated that melanin obtained from hiPSC-Mel had the lowest photoreactivity among the tested natural pigments, indicating the photoprotective potential.

## 2. Results and Discussion

### 2.1. Paramagnetic Properties of the Examined Melanins

**Figure 1:**
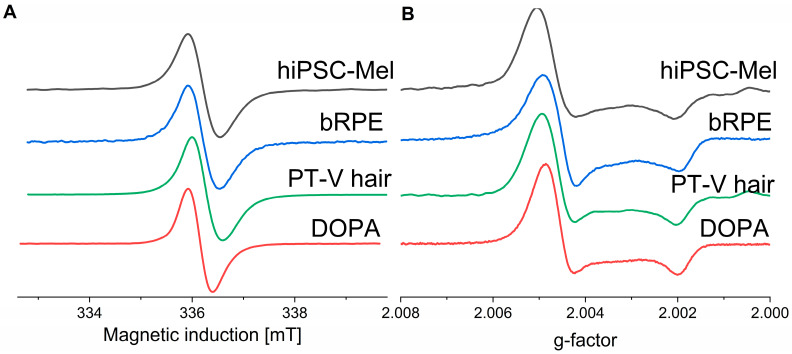
Paramagnetic properties of melanins, under zinc ion saturating conditions. obtained with X-band (**A**) and W-band (**B**) EPR spectroscopy.

To determine the paramagnetic properties of the melanins, electron paramagnetic resonance (EPR) spectroscopy at 9.4 GHz (X-band) and 94 GHz (W-band) was employed. X-band EPR spectra of the melanins are shown in Figure 1A. All melanins exhibited similar EPR signals consisting only of a single line characteristic for pigments mostly of eumelanin origin. To verify these results, W-band EPR spectroscopy, operating at ten-fold higher microwave frequency, was used and the results are shown in Figure 1B. The spectra are consistent with a substantial axial symmetry of the melanin paramagnetic centers. It is apparent that both low- and high-field components of the spectra of all natural melanins exhibit g-values very similar to that of the synthetic DOPA-melanin [40,41]. The fact that the low-field line at *g* around 2.005 was slightly broader for natural melanins, compared to synthetic DOPA-melanin, could be attributed to higher inhomogeneity of the radicals of natural melanins and/or possible contamination of the natural pigment with paramagnetic metal ions. Similar results were obtained previously for natural melanins [13].

### 2.2. Chemical Analysis of Melanin Degradation Products

**Figure 2:**
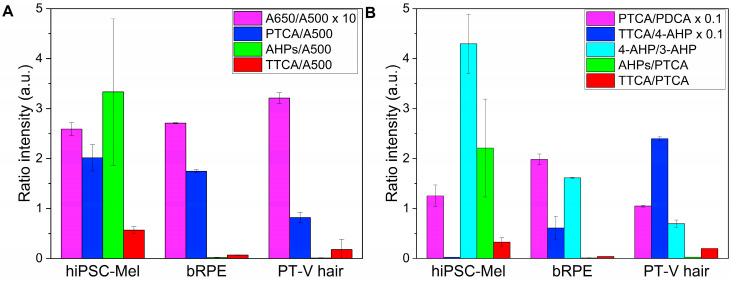
Ratios of the examined markers for the examined melanins (**A,B**).Values represent mean ± SEM. A650/A500 ratios for all samples are multiplied by a factor of 10. PTCA/PDCA and TTCA/4-AHP for all samples are multiplied by a factor of 0.1 to increase clarity.

To determine the chemical composition of the melanins, alkaline hydrogen peroxide oxidation (AHPO) [42] and hydroiodic acid (HI) hydrolysis [43] were employed. Ratios of the selected markers obtained by chemical degradation of the examined melanins are shown in Figure 2, while the specific values of the markers are shown in Table 1 and Table 2. As the exact amount of melanin could not be accurately measured gravimetrically due to the low quantity of the pigment in the samples, the amounts of the pigments are assessed by comparing their normalized absorbance at 500 nm (A500). The A500 value obtained by melanin solubilization with Soluene-350 reflects the total amount of melanin [44]. The A650/A500 ratio indicates whether melanin is eumelanic (ratio > 0.20) or pheomelanic (<0.15). AHPO affords the determination of pyrrole-2,3,5-tricarboxylic acid (PTCA), pyrrole-2,3-dicarboxylic acid (PDCA) and thiazole-2,4,5-tricarboxylic acid (TTCA) as markers of 5,6-dihydroxyindole-2-carboxylic acid (DHICA) and 5,6-dihydroxyindole (DHI) moieties of eumelanin and benzothiazole (BZ) moiety of pheomelanin [42]. HI hydrolysis yields 4-amino-3-hydroxyphenylalanine (4-AHP) and 3-amino-4-hydroxyphenylalanine (3-AHP), which are markers of 5-*S*-cysteinyldopa- and 2-*S*-cysteinyldopa-derived benzothiazine (BT) moieties, respectively [43,44]. A comparison of the A650/A500 ratios suggested that hiPSC melanin was mainly eumelanic, as were the other two natural melanins, bRPE melanin, and PT-V hair melanin. However, the high AHP (4-AHP + 3-AHP)/A500 ratio indicated that hiPSC melanin contained more BT-pheomelanin than the others, consistent with the high AHPs/PTCA ratio. A unique feature of hiPSC melanin was the low TTCA/4-AHP ratio (an indicator of BZ/BT ratio; 0.25), compared with those in bRPE melanin (6.11) and PT-V melanin (24.0). The data suggest that bRPE melanin and PT-V melanin were possibly affected by photoaging, which could induce photochemical modifications in the pheomelanic moiety [45]. The proposed photoaging is consistent with the lower AHPs/PTCA, 4-AHP/3-AHP, TTCA/PTCA ratios, and the higher TTCA/4-AHP ratios in bRPE and PT-V melanins. The PTCA/PDCA ratio (an indicator of DHICA/DHI ratio) was similar for all examined melanins. Note that the conversion factors of PTCA, 4-AHP and TTCA are 38, 9 and 34, respectively [46].

### 2.3. Size of Melanosomes and Nanoaggregates

**Figure 3:**
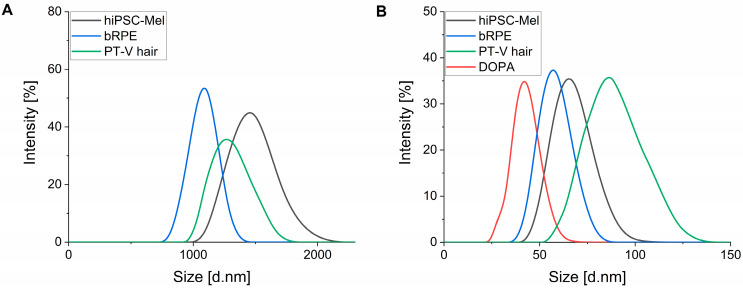
Size distribution of melanosomes (**A**) and nanoaggregates (**B**) obtained for the examined melanins.

The size distribution of melanosomes and nanoaggregates was obtained using dynamic light scattering (DLS) and the relevant results are shown in Figure 3. Compared to bRPE and PT-V hair melanin, the diameter of hiPSC-Mel was the largest. The average size of melanosomes from hiPSC-Mel was around 1.49 ± 0.28 μm; bRPE melanosomes showed a diameter of around 1.10 ± 0.21 μm, whilst pigment granules from black human hair demonstrated an average diameter of 1.28 ± 0.21 μm (Figure 3A). These results compare reasonably well with the previously reported size of skin melanosomes found in phototype V/VI (1.44 ± 0.67 μm) determined by electron micrographs [48], and DLS measurements of isolated bRPE melanosomes, showing an average diameter of 0.94 ± 0.20 μm [49]. Nanoaggregates obtained from hiPSC-Mel, bRPE and PT-V hair exhibited a diameter of around 68 ± 12 nm, 59 ± 10 nm, and 86 ± 18 nm, respectively. These values were slightly larger than the particle diameters determined for of synthetic DOPA-melanin (38 ± 7 nm) (Figure 3B).

### 2.4. Photoconsumption of Oxygen by the Examined Melanins

**Figure 4:**
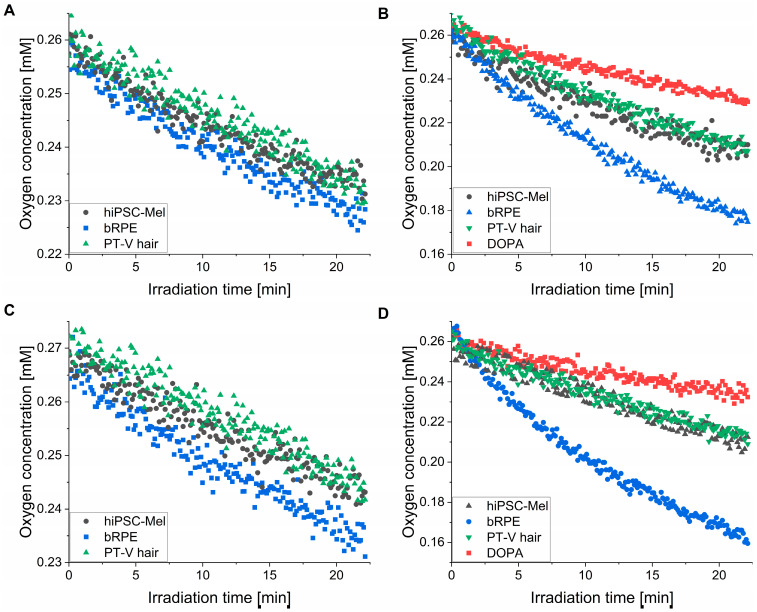
Photoinduced consumption of oxygen by the examined melanins under 365 nm (4.5 mW/cm^2^) (**A**,**B**) and 445 nm (13.8 mW/cm^2^) (**C**,**D**) irradiation. (**A**,**C**) Demonstrate results obtained for melanosomes, whereas (**B**,**D**) show results for melanin nanoaggregates and DOPA-melanin.

EPR oximetry was used to determine the ability of the examined melanosomes and nanoaggregates to photoconsume oxygen (Figure 4). It is evident that during irradiation with 365 nm (Figure 4A,B) and 445 nm light (Figure 4C,D), all melanins induced the steady depletion of dissolved oxygen with the highest rate of oxygen photoconsumption observed for bRPE melanin. The determined rates of the initial oxygen photoconsumption are shown in Table 3.

The rates of oxygen consumption were considerably higher for nanoaggregates than for melanosomes: at 365 nm, the corresponding rates were 2.4–4.4-fold higher and at 445 nm they were 1.9–5.9-fold higher (Table 3). This could be attributed to the smaller size of the nanoaggregates compared to melanosomes. The resulting higher surface-to-volume ratio for the nanoaggregates contributes to an increased accessibility of the melanin functional groups to external agents. Although only a small difference in the ability of different melanosomes to photoconsume oxygen was observed, the corresponding photoreactivity of nanoaggregates from bRPE was significantly higher compared to nanoaggregates from the other natural melanins. Furthermore, photoconsumption was approximately 2–4 times more efficient under UVA compared to visible light irradiation. Synthetic DOPA melanin, in spite of a similar size of its particles compared to nanoaggregates from natural melanins, demonstrated significantly lower rates of oxygen photoconsumption. It should be stressed that the photoconsumption of oxygen is a non-specific method used for measuring aerobic photoreactivity. Although it does not provide any direct information about the nature of the reaction, this method is fairly sensitive and was previously used successfully for the characterization of melanin’s photochemistry [13,14,17].

### 2.5. Photoproduction of Superoxide Anion by the Examined Melanins

**Figure 5:**
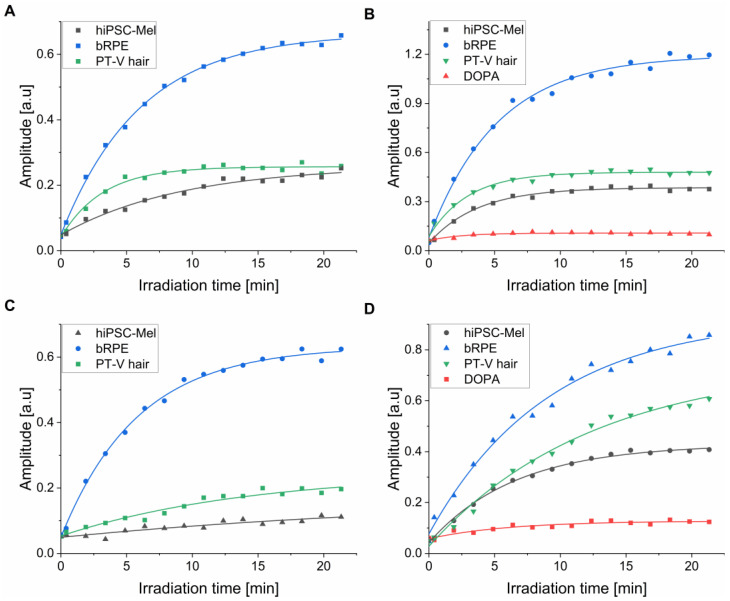
Kinetics of DMPO-OOH spin adduct formation during 365 nm (4.5 mW/cm^2^) (**A**,**B**) and 445 nm (13.8 mW/cm^2^) (**C**,**D**) light irradiation.

EPR spin-trapping was employed to measure the kinetics of the type I photosensitized reaction, in which superoxide anion was produced. Figure 5 shows the accumulation of DMPO-OOH spin adduct, resulting from the interaction of the spin trap with superoxide anion generated by melanosomes (Figure 5A,C) and nanoaggregates (Figure 5B,D) upon irradiation with UVA (Figure 5A,B) or with blue light (Figure 5C,D). Samples of melanins for EPR-spin trapping were prepared in 70%/30% DMSO/water mixture to facilitate the detection of the DMPO-OOH spin adduct. Importantly, the presence of aprotic solvent extends the lifetime of the spin adduct [13,50]. The initial growth of the DMPO-OOH signal, which can be attributed to the efficacy of the pigments to photoproduce superoxide anion, was considerably higher for nanoaggregates (Figure 5B,D) than for melanosomes (Figure 5A,C). The calculated initial rates of the DMPO-OOH adduct accumulation are shown in Table 2. Similar to oxygen photoconsumption, the photoproduction of superoxide anion was more efficient during the irradiation of melanin with UVA than with visible light (Table 4). The formation of superoxide anion was found to be significantly lower for hiPSC-Mel compared to melanin obtained from bRPE with 4.4 to 23 lower rates for melanosomes and around 1.7 to 2.3 for melanin nanoaggregates. Also melanosomes and nanoaggregates from PT-V hair showed higher efficiency for photogenerating superoxide anion (2.9–3.3 times and up to 1.4 times, respectively) than melanin from hiPSC-Mel. The observed difference in the rate of superoxide anion photoproduction, particularly when comparing nanoaggregates, is consistent with their ability to photoconsume oxygen. Melanosomes from hiPSC-Mel demonstrated low efficiency in photogenerating superoxide anion, comparable to synthetic DOPA-melanin, which is substantially lower than that of other natural melanins.

### 2.6. Generation of Singlet Oxygen by the Examined Melanins

**Figure 6:**
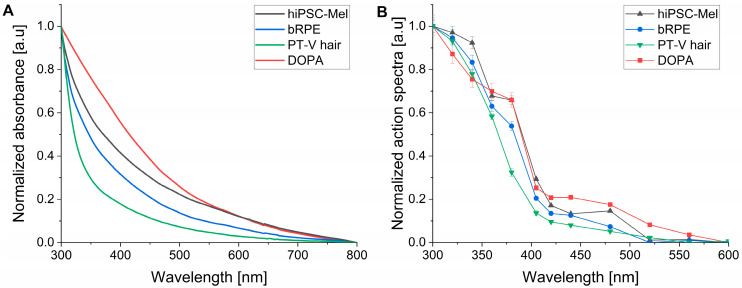
Normalized UV–Vis absorption spectra (**A**) and normalized action spectra of singlet oxygen photogeneration (**B**) by nanoaggregates of the examined melanins.

Quantum yields (Φ_Δ_) of singlet oxygen generation at 365 nm and 445 nm excitation and action spectra for singlet oxygen photogeneration were measured using time-resolved phosphorescence at 1270 nm. The formation of singlet oxygen via a Type II photosensitized reaction requires energy transfer from a relatively long-lived triplet excited state of a photosensitizer molecule to ground state molecular oxygen [51]. Although based on ultrafast spectroscopy experiments, the formation of triplet excited states of melanin was ruled out by most researchers [52], while some studies suggested that photoexcitation of melanin could lead to the generation of reactive intermediates including triplet excited states [53,54,55,56,57]. Prior to singlet oxygen measurements, UV–Vis absorption spectra of the examined melanins were determined (Figure 6A). Compared to bRPE and PT-V hair melanins, the pigment from hiPSC-Mel demonstrated higher absorbance in both UV and visible parts of the spectrum consistent with its postulated lower photodegradation. Action spectra of singlet oxygen photogeneration for the tested melanins are shown in Figure 6B. It is apparent that photogeneration of singlet oxygen increases with decreasing wavelength. A particularly significant increase is observed between 420 and 340 nm which is in agreement with previously reported data for melanins from human hair samples [13]. The obtained action spectra for the photogeneration of singlet oxygen by the pigments generally followed their absorption suggesting that the main melanin chromophores might be responsible for the photogeneration of singlet oxygen.

**Figure 7:**
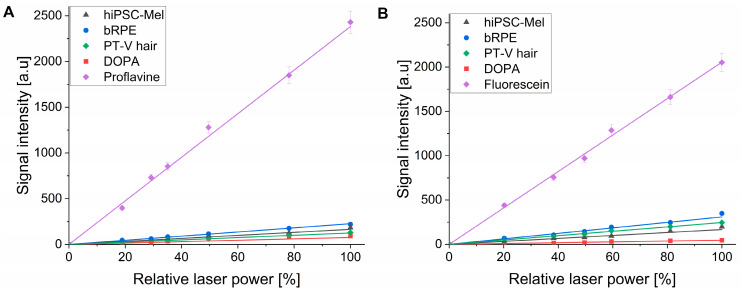
Determination of quantum yield of singlet oxygen photogeneration by the examined melanins under 365 nm (**A**) and 445 nm (**B**) excitation.

Although action spectra for singlet oxygen generation provide qualitative characterization of the photoreactive properties of a photosensitizer at different wavelengths, quantitative characterization requires the determination of quantum yield of singlet oxygen photogeneration (Φ_Δ_), at least at selected wavelengths. The results of such a determination are shown in Figure 7. Quantum yields were determined for two excitation wavelengths: 365 nm (Figure 7A) and 445 nm (Figure 7B) using a comparative method and employing two standards: proflavine (Φ_Δ_ = 0.108 measured against Rose Bengal) for 365 nm excitation and fluorescein (Φ_Δ_ = 0.035 measured against Rose Bengal) for 445 nm excitation.

The calculated values of Φ_Δ_ gathered for different melanins in Table 5 show that the efficiency of the examined melanin to generate singlet oxygen was about two-fold higher at 365 nm than at 445 nm. The melanin pigment from bRPE was found to photogenerate singlet oxygen 1.6 to 1.9 times more efficiently than melanin obtained from hiPSC-Mel, consistent with the results of oxygen photoconsumption. Also, melanin from human hair was found to generate singlet oxygen with 1.3–1.8 times higher yield than hiPSC melanin. Quantum yields of singlet oxygen photogeneration were found to be of the same order of magnitude for hiPSC-Mel, bovine RPE and PT-V hair melanin. The data are in agreement with previously obtained data regarding melanins obtained from hair [13].

## 3. Materials and Methods

### 3.1. Generation of Melanocytes from hiPSCs

Melanocytes were generated from protein-induced pluripotent stem cells using a multistep protocol described in detail elsewhere [34]. The protocol was further modified to obtain highly pigmented cells based on the following work [38]. In brief, protein-induced pluripotent stem cells (piPS) (System Biosciences, Palo Alto, CA, USA) were cultured on a feeder layer of mouse embryonic fibroblasts in DMEM/F12 culture media supplemented with 20% KSR (both from ThermoFisher Scientific, Waltham, MA, USA), 100 µM β-mercaptoethanol, 2 mM GLUTAMAX (Sigma-Aldrich, St. Louis, MO, USA), 100 µM non-essential amino acids, 100 U/mL/100 µg/mL penicillin/streptomycin, and 10 ng/mL bFGF (all from ThermoFisher Scientific, Waltham, MA, USA) until the confluency was reached. To induce embryoid body (EB) formation, cells were washed with PBS, dissociated by Accutase (Thermofisher Scientific, Waltham, MA, USA) and plated at 2.5–5 × 10^4^ cells/cm^2^ in above-described medium supplemented with Y27632 and without bFGF. After 4 days, when EBs were formed, cells were plated onto an adhesive dish and cultured in DMEM supplemented with 10% FBS, 4.85 g/L glucose, 2 mM L-glutamine and 100 U/mL/100 µg/mL penicillin/streptomycin. To induce differentiation into melanocytes, developed differentiation media were used.

### 3.2. Isolation of Melanin from hiPSC-Mel

Melanocytes derived from hiPSCs were incubated in PBS: PBS + 1 mM EDTA (Sigma-Aldrich, St. Louis, MO, USA) (1:1) and then transferred into a glass tissue grinder with a PTFE pestle. Cells were homogenized in 5–10 cycles consisting of 100 downward and upward moves each. After each cycle, cells were centrifuged for 5 min (60× *g*, 4 °C), the supernatant was collected and transferred into a separate centrifuge tube, and the remaining pellet was resuspended in PBS: PBS + 1 mM EDTA (1:1). Finally, the collected supernatant was centrifuged for 10 min (5500× *g*, 4 °C), the remaining supernatant containing melanin nanoaggregates was transferred to a separate tube, and the obtained pellet containing isolated melanosomes was suspended in PBS (pH = 7.4).

### 3.3. Isolation of Melanin from Human Hair

Natural melanin pigment was obtained from human hair samples generously donated by one black individual from Africa, non-smoker, living in a mid-European country. Melanosomes and melanin nanoaggregates were isolated according to the protocol described in detail elsewhere [13].

### 3.4. Isolation of Melanin from Bovine Retinal Pigment Epithelium

Melanosomes and melanin nanoaggregates were isolated from the RPE of bovine eyes using the protocol described in detail elsewhere [58]. In brief, melanosomes were purified from the RPE of bovine eyes and purified granules were incubated in Laemmli electrophoresis buffer containing protease inhibitors to remove contaminating materials and proteins or membranes associated with the granule surface.

### 3.5. Preparation of Synthetic Melanin

Synthetic model of eumelanin prepared by the autooxidation of DOPA was synthesized and purified using a protocol described in detail elsewhere [47]. Briefly, 25 g of DL-DOPA (Sigma-Aldrich, St. Louis, MO, USA) was transferred into 5 L of water, alkalized to pH 8 using 25% ammonia solution and left for 3 days while bubbled with air and magnetically stirred. The pH of the mixture was adjusted to pH 8 every few hours. For the following 3 days, the solution was further stirred without air bubbling. Finally, the solution was acidified to pH 2.5, washed at 4040× *g* for 10 min. The obtained pellet was 8 times washed with 4 L of acidified water (pH 2.5) and once with nonacidified water (pH 5.5). The precipitate was resuspended in 0.5 L and dialyzed for 12 days against 5 L of water changed every 3 days. To determine the final concentration of the melanin, dry mass determination was performed. Briefly, three independent 0.2 mL samples of the final solution were dried under vacuum and weighed. Based on the measurements, the final yield of the synthesis was calculated as approximately 20%.

### 3.6. X-Band Electron Paramagnetic Resonance Spectroscopy Measurements

EPR examination of iPSC-derived melanin, bovine RPE, PT-V hair melanin and DOPA-melanin in 50 mM zinc acetate was carried out at 77K using a quartz finger-type dewar, employing Bruker EMX-AA EPR spectrometer (Bruker BioSpin, Rheinstetten, Germany) using the settings described elsewhere [13].

### 3.7. W-Band Electron Paramagnetic Resonance Spectroscopy Measurements

W-band EPR spectra were collected using a spectrometer system constructed at the National Biomedical EPR Center at Medical College of Wisconsin, USA. Samples placed in 0.2 mm ID and 0.33 OD synthetic silica capillaries (Fiber Optic Center, New Bedford, MA, USA) were measured in the cylindrical TE011 cavity resonator, specially designed for aqueous samples operating at 94.04 GHz [59,60]. During the measurement, sample and resonator were maintained at 25 °C using the temperature-controlled water bath circulating through a clamp attached to the resonator assembly. The samples were examined in 20 mM acetate buffer using the following parameters: 50 µW microwave power, 937 Hz field modulation frequency, 0.4 mT peak-to-peak modulation amplitude, 20 mT magnetic field scan width. The obtained spectra were the result of multiple scans averaged over a period as required for adequate signal-to-noise ratio (20–40 min) [17].

### 3.8. Chemical Analysis of Melanin Subunits

A500 (absorbance at 500 nm) and A650 values were measured using Soluene-350 solubilization [44]. Pyrrole-2,3-dicarboxylic acid (PDCA), pyrrole-2,3,5-tricarboxylic acid (PTCA), and thiazole-2,4,5-tricarboxylic acid (TTCA), which are the markers of 5,6-dihydroxyindole (DHI), 5,6-dihydroxyindole-2-carboxylic acid (DHICA) eumelanin subunits, native and modified benzothiazole units of pheomelanin, respectively, were measured using alkaline H_2_O_2_ oxidation (AHPO) as described elsewhere [42]. These markers were analyzed by the improved method of HPLC [61]. The amounts of 4-amino-3-hydroxyphenylalanine (4-AHP) and 3-amino-4-hydroxyphenylalanine (3-AHP), which are the markers of 5-*S*-cysteinyldopa and 2-*S*-cysteinyldopa, respectively, were measured using hydroiodic acid (HI) hydrolysis as described elsewhere [43].

### 3.9. UV–Vis Absorption Spectroscopy

The optical absorption of natural melanins and DOPA-melanin was measured in D_2_O (Sigma-Aldrich, Saint Louis, MO, USA) using HP 8453 diode array spectrophotometer (Hewlett-Packard, Palo Alto, CA, USA). The obtained spectra were corrected by the subtraction of the value at 800 nm from the values at lower wavelengths and normalized in the range 300–800 nm.

### 3.10. Size Analysis

The size of melanin pigments was determined by the dynamic light scattering (DLS) technique, using a Malvern Zetasizer Nano S (Malvern Panalytical, Malvern, UK) as described elsewhere [13]. Samples were diluted in filtered water to a concentration of about 0.01 mg mL^−1^.

### 3.11. Oxygen Consumption Measurements

Time-dependent changes in oxygen concentration induced by light were determined by electron paramagnetic resonance (EPR) oximetry using mHCTPO (4-protio-3-carbamoyl-2,2,5,5-tetraperdeuteromethyl-3-pyrrolin-1-yloxy) at 0.1 mM concentration as dissolved oxygen-sensitive spin probe. Samples containing 0.15 mg/mL of melanins in PBS (pH = 7.4) were irradiated in EPR quartz flat cell in the resonant cavity using 365 nm light (4.5 mW/cm^2^) or 445 nm light (13.8 mW/cm^2^) generated by LED light chips (High Power LED Chip, Chanzon, Shenzhen, China) as described previously [13].

### 3.12. Electron Paramagnetic Resonance Spin Trapping Studies

EPR spin-trapping of photogenerated superoxide anion was performed using 100 mM DMPO (5,5-dimethyl-1-pyrroline N-oxide) (Sigma-Aldrich, Saint Louis, MO, USA) as a spin trap, according to the procedure described elsewhere [13]. Samples containing 0.075 mg/mL melanin in 70% DMSO/30% water (POCH, Gliwice, Poland) were irradiated using the same light source as described for oxygen photoconsumption (*vide supra*), employing parameters described elsewhere [14].

### 3.13. Time-Resolved Singlet Oxygen Phosphorescence

Formation and decay of singlet oxygen phosphorescence were measured in phosphate-buffered D_2_O (pH = 7) according to the procedure described elsewhere [17]. Samples in a 10 mm optical path quartz fluorescence cuvette (QA-1000, Hellma, Mulheim, Germany) were excited with light pulses generated by an integrated nanosecond DSS Nd: YAG laser system equipped with a narrow-bandwidth optical parameter oscillator (NT242-1k-SH/SFG, Ekspla, Vilnius, Lithuania). Photoexcited generation of singlet oxygen by melanin nanoaggregates was examined in the spectral range of 300–600 nm. Quantum yield of singlet oxygen photogeneration was determined for 365 nm, and 445 nm excitation using a comparative method described elsewhere [13]. The absorbance of melanins and employed photosensitizers was adjusted to 0.150 ± 0.005 for 365 nm excitation, and to 0.100 ± 0.004 for 445 nm excitation. To adjust photoexcitation energy in experiments designed to determine the quantum yield of singlet oxygen photogeneration, the laser beam was attenuated with 1–3 pieces of wire mesh (light transmission of each piece ~30%). All samples were constantly stirred using a dedicated magnetic stirrer. The 1270 nm luminescence was measured, and the data were collected, using the system described elsewhere [13].

### 3.14. Statistical Analysis

All experiments were performed at least three times. Statistical analysis of the data was performed using ANOVA with a post hoc Tukey test employing OriginPro Software version 2024 (OriginLab, Northampton, MA, USA).

## 4. Conclusions

In this work, we examined key physicochemical parameters, including the aerobic photoreactivity of melanin isolated from hiPSC-Mel, and compared them with melanin from other known sources of the pigment, such as bovine retinal pigment epithelium (bRPE) and phototype V (PT-V) hair. Electron paramagnetic resonance (EPR) spectroscopy, dynamic light scattering, UV–Vis absorption and HPLC analysis of melanin degradation products were used. The ability of the examined melanins to photogenerate reactive oxygen species was determined, using EPR oximetry, EPR spin-trapping and time-resolved singlet oxygen phosphorescence. Although the results of such measurements demonstrated that the melanin obtained from hiPSC-Mel exhibited physicochemical properties typical for eumelanin, a contribution from pheomelanin with a substantial presence of benzothiazine subunits, was also evident. Importantly, the hiPSC-Mel pigment had significantly lower photoreactivity compared to bRPE melanin and PT-V hair melanin. Our findings indicate that hiPSC-Mel could be an excellent source of high-quality pigment for photoprotection studies.

## Figures and Tables

**Table 1:** Selected parameters of examined melanins normalized to melanin absorbance at 500 nm.

Origin of the Pigment	A650/ A500 ^1^	PTCA (µg)/ A500 ^1^	AHPs (µg)/ A500 ^1^	TTCA (µg)/ A500 ^1^
hiPSC-Mel	0.259 ± 0.013	2.015± 0.263	3.333 ± 1.465	0.566 ± 0.072
bRPE	0.271 ± 0.001	1.747 ± 0.030	0.021 ± 0.006	0.069 ± 0.005
PT-V hair	0.321 ± 0.001	0.819 ± 0.106	0.010 ± 0.008	0.178 ± 0.205
DOPA-mel ^2^	0.321 ± 0.002	0.340 ± 0.007	0.000 ± 0.000	0.000 ± 0.000

^1^ Data represent mean ± SEM. ^2^ Data previously reported by the authors [47].

**Table 2:** Ratio of determined melanin markers.

Origin of the Pigment	PTCA/ PDCA ^1^	TTCA/ 4-AHP ^1^	4-AHP/ 3-AHP ^1^	AHPs/ PTCA ^1^	TTCA/ PTCA ^1^
hiPSC-Mel	12.539 ± 2.179	0.252 ± 0.033	4.295 ± 0.59	2.210 ± 0.981	0.327 ± 0.087
bRPE	19.821 ± 1.089	6.111 ± 2.265	1.613 ± 0.013	0.012 ± 0.004	0.040 ± 0.002
PT-V hair	10.486 ± 0.020	23.980 ± 0.357	0.697 ± 0.07	0.028 ± 0.001	0.202 ± 0.001
DOPA-mel ^2^	2.104 ± 1.234	0.000 ± 0.000	0.000 ± 0.000	0.000 ± 0.000	0.000 ± 0.000

^1^ Data represent mean ± SEM. ^2^ Data previously reported by the authors [47].

**Table 3:** Initial rates of oxygen photoconsumption obtained for the examined melanins under 365 nm and 445 nm irradiation.

Origin of the Pigment		Oxygen Consumption at 365 nm [nM (O_2_)/s] ^1^	Oxygen Consumption at 445 nm [nM (O_2_)/s] ^1^
hiPSC-Mel	Melanosomes	60.47 ± 3.20	19.00 ± 1.26
bRPE	Melanosomes	67.71 ± 4.52	23.67 ± 2.87
PT-V hair	Melanosomes	64.09 ± 2.29	21.67 ± 2.32
hiPSC-Mel	Nanoaggregates	142.65 ± 8.92	38.83 ± 4.55
bRPE	Nanoaggregates	297.71 ± 19.33	139.33 ± 11.79
PT-V hair	Nanoaggregates	160.74 ± 5.35	40.17 ± 3.79
DOPA-melanin		77.53 ± 8.92	20.16 ± 2.64

^1^ Data represent mean ± SD.

**Table 4:** Initial rates of superoxide anion adduct formation for the examined melanins under 365 nm and 445 nm irradiation.

Origin of the Pigment		Rate of DMPO-OOH Generation at 365 nm [a.u × 10^−4^/s] ^1^	Rate of DMPO-OOH Generation at 445 nm [a.u × 10^−4^/s] ^1^
hiPSC-Mel	Melanosomes	11.87 ± 1.21	0.67 ± 0.13
bRPE	Melanosomes	52.56 ± 4.62	15.60 ± 1.22
PT-V hair	Melanosomes	34.91 ± 3.39	2.24 ± 0.11
hiPSC-Mel	Nanoaggregates	47.24 ± 4.20	9.59 ± 1.23
bRPE	Nanoaggregates	110.22 ± 8.97	16.65 ± 2.22
PT-V hair	Nanoaggregates	67.98 ± 5.24	9.75 ± 1.05
DOPA-melanin		9.85 ± 1.11	1.91 ± 0.21

^1^ Data represent mean ± SD.

**Table 5:** Quantum yields of singlet oxygen photogeneration at two different wavelengths for the examined melanins.

Origin of the Pigment	Quantum Yield at 365 nm [%] ^1^	Quantum Yield at 445 nm [%] ^1^
hiPSC-Mel	0.53 ± 0.05	0.28 ± 0.02
bRPE	0.95 ± 0.13	0.53 ± 0.03
PT-V hair	0.69 ± 0.09	0.42 ± 0.07
DOPA-melanin	0.16 ± 0.03	0.08 ± 0.01

^1^ Data represent mean ± SD.

## Data Availability

The original contributions presented in this study are included in the article. Further inquiries can be directed to the corresponding author.
